# Development and characterization of a DNA aptamer for MLL-AF9 expressing acute myeloid leukemia cells using whole cell-SELEX

**DOI:** 10.1038/s41598-021-98676-4

**Published:** 2021-09-27

**Authors:** Kaylin G. Earnest, Erin M. McConnell, Eman M. Hassan, Mark Wunderlich, Bahareh Hosseinpour, Bianca S. Bono, Melissa J. Chee, James C. Mulloy, William G. Willmore, Maria C. DeRosa, Edward J. Merino

**Affiliations:** 1 Department of Chemistry, University of Cincinnati, Cincinnati, OH USA; 2 Department of Chemistry, Carleton University, Ottawa, ON Canada; 3 Division of Experimental Hematology and Cancer Biology, Cincinnati Children’s Hospital Medical Center, Cincinnati, OH USA; 4 Department of Neuroscience, Carleton University, Ottawa, ON Canada

**Keywords:** Cancer, Analytical chemistry, DNA

## Abstract

Current classes of cancer therapeutics have negative side effects stemming from off-target cytotoxicity. One way to avoid this would be to use a drug delivery system decorated with targeting moieties, such as an aptamer, if a targeted aptamer is available. In this study, aptamers were selected against acute myeloid leukemia (AML) cells expressing the MLL-AF9 oncogene through systematic evolution of ligands by exponential enrichment (SELEX). Twelve rounds of SELEX, including two counter selections against fibroblast cells, were completed. Aptamer pools were sequenced, and three candidate sequences were identified. These sequences consisted of two 23-base primer regions flanking a 30-base central domain. Binding studies were performed using flow cytometry, and the lead sequence had a binding constant of 37.5 + / − 2.5 nM to AML cells, while displaying no binding to fibroblast or umbilical cord blood cells at 200 nM. A truncation study of the lead sequence was done using nine shortened sequences, and showed the 5′ primer was not important for binding. The lead sequence was tested against seven AML patient cultures, and five cultures showed binding at 200 nM. In summary, a DNA aptamer specific to AML cells was developed and characterized for future drug-aptamer conjugates.

## Introduction

Among the various types of leukemia, acute myeloid leukemia (AML) has a poor prognosis. As of 2016, AML has a 5-year survival rate of only a 26%^1^. By the end of 2020, there will be an estimated 19,940 new cases of AML, and an estimated 11,180 deaths from the disease in the United States^2^. The current treatment for most types of AML is chemotherapy. From the first chemotherapeutic experiments 60 years ago, researchers have worked to develop more effective cancer drugs^3^. Advances in understanding of the molecular and cellular biology of cancers have allowed specific targeting of signaling proteins and pathways involved in cancer initiation and progression^4,5^. However, current classes of cancer therapeutics, including those used to treat AML (i.e. small molecule drugs such as doxorubicin), are riddled with negative side effects, stemming from off-target cytotoxicity, a major problem in cancer drug design, because of the lack of selectivity of these therapeutics toward cancer cells^6–11^.

A further challenge in treating cancer is the inconsistent efficacy of anticancer agents, which are sometimes promising in vitro yet not always suitable for use in vivo. Some of the main pharmacological concerns are aqueous solubility, stability, and bioavailability^12,13^. Many of the pharmacological properties of free, non-conjugated, anticancer agents can be improved by using drug delivery systems (DDS). These DDS are designed to alter the pharmacokinetic properties of therapeutics to increase solubility and stability, thereby altering the effective bioavailability and biodistribution^14–16^. One effective strategy for drug delivery is the conjugation of a therapeutic to a targeting moiety. The development of this type of DDS could significantly improve cancer patient management.

A new rising class of targeting agents for use as a component of potential DDS is aptamers. Aptamers possess several advantages over other ligands typically used in drug delivery such as antibodies. Aptamers are single-stranded oligonucleotides that are selected through an iterative in vitro process called systematic evolution of ligands by exponential enrichment (SELEX) to bind to specific targets with high affinity and selectivity^17–20^. A random library of DNA or RNA ligands are subjected to sequential binding experiments, so that after a series of partitioning cycles, a single ligand will emerge^18^. SELEX relies on repeating positive and negative selection processes that eliminate weakly binding and non- binding sequences. Targets can be small molecules, proteins, or even whole cells^21,22^. Aptamers have quickly emerged as a novel and powerful class of ligands with excellent potential for diagnostic and therapeutic applications, particularly in cancer-based applications^23,24^. In fact, the usability of aptamers in cancer applications such as biomarker discovery, the development of diagnostic imaging and technology, therapeutic and theranostic tools, and as targeting agents have recently been reviewed^25–30^. Unlike antibodies, aptamers can be synthesized without relying on biological systems, which makes them easier to produce with less batch-to-batch variability^31–34^. Also, they are quite thermally stable and can be denatured and renatured several times without significant loss of activity^35,36^. Aptamers are also smaller than antibodies, which can lead to better tissue penetration in solid tumors^37,38^. Additionally, the lack of the ability to induce an immune response is another favorable advantage of aptamers over antibodies^37,39^. Though aptamers are different from antibodies, they mimic properties of antibodies in a variety of diagnostic formats. Conjugation of functional groups to aptamers is facile thanks to advancements in nucleic acid chemistry, which means they can be introduced during synthesis, a particularly favorable advantage over antibodies which generally require post-synthetic modifications^20,40^. Also, the addition of labeling groups, such as fluorescent dyes, expands the potential application of these functional nucleic acids in cancer applications, and show particular potential in therapeutic development^41,42^.

Aptamer-drug conjugates can enable targeted therapy, which is a way to reduce adverse side effects and potentially increase toxicity to the specific cells, and are being used in chemotherapy, gene therapy, immunotherapy, photodynamic therapy, and photothermal therapy, primarily of cancer^43–46^. For example, doxorubicin conjugated to a protein tyrosine kinase 7 (PTK7) aptamer has selectively inhibited cancer cell proliferation in T-cell acute lymphoblastic leukemia^47^. The same aptamer was conjugated to 5-fluorouracil to treat colon cancer cells^48^. Moreover, methotrexate, a central drug used in AML chemotherapy regimens, has been conjugated to a CD117 (a biomarker highly expressed on AML cells) specific aptamer, and the methotrexate-aptamer conjugate specifically inhibited AML cell growth and triggered cell apoptosis with little effect on CD117-negative cells^49^.

A modification of the traditional SELEX process that uses living whole cells as targets, as opposed to a specific target ligand, has been proven^50,51^ and is known as cell-SELEX^52^. Cell-SELEX has an advantage over traditional SELEX, because cell-SELEX targets proteins expressed on cell surfaces in their native state rather than purified recombinant proteins; therefore, cell-SELEX is more desirable for the selection of aptamers to target cancer cells^53,54^. Aptamers with high affinity and specificity for cells have been produced successfully, demonstrating that complex targets, including tumor cells and tissues, are compatible with the SELEX process^24,55–57^. In this manuscript, we utilize a developed cellular model of AML where the leukemia-associated oncogene MLL-AF9 is expressed in human blood stem/progenitor cells^58^. The mixed lineage leukemia gene, MLL, found at chromosome band 11q23 is regularly involved in reciprocal translocations in acute leukemias, and the MLL fusion genes contribute to leukemogenesis; the AF9 protein, a transcriptional activator, is a common MLL fusion partner in AML^59^. The molecular signatures associated with these AML cells closely mimic those identified in primary AML patient samples^60^. This model was used as a target to develop a DNA aptamer through a cell-SELEX process. Thus, binding experiments between this novel model, blood stem cells, primary patient samples, and fibroblast cells accurately reflect relative specificity of our aptamer.

## Results

### Development of aptamers that bind specifically to MLL-AF9 RAS cells

**Figure 1:**
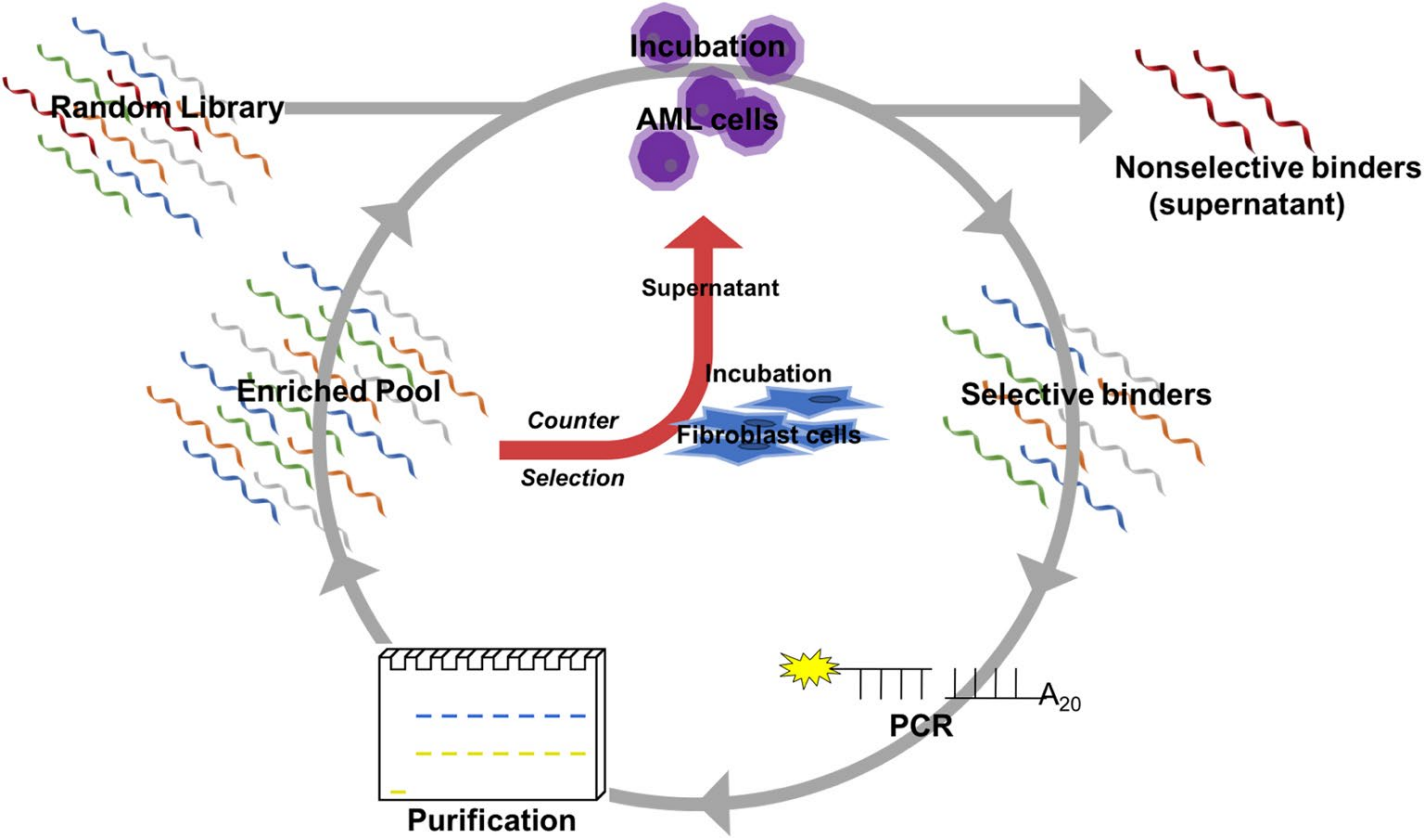
Schematic diagram of SELEX method for selection of MLL-AF9 RAS-specific DNA aptamers with internal counter selection method.

Whole cell-based SELEX was effectively used for selection, using MLL-AF9 RAS (MA9Ras) AML cells as the positive selection target and fibroblast connective tissue cells as the counter selection. Using a connective tissue as a counter target was done as a preventative tactic to have the aptamer remain in the blood stream to better find its target cells upon application in an animal model. The reaction schematic is shown in Fig. 1.

**Figure 2:**
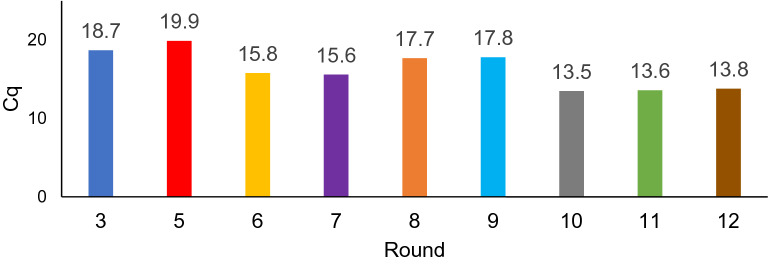
Monitoring enrichment of the ssDNA library of MLL-AF9 RAS during SELEX by qPCR.

A summary schematic of the SELEX method workflow is shown in Figure S1. Briefly, a single-stranded DNA library made up of a template that had a 30 base randomized region flanked by known 5′- and 3′- primer regions was subjected to consecutive binding and elution to enrich DNA sequences specific to the target cell. Selection was done by heating the DNA pool to 95 °C. Once cooled to room temperature, one million cells were suspended in the DNA pool-buffer solution and incubated. After incubation, cells were pelleted, and the supernatant was removed and discarded to partition non-binding sequences. Cells were resuspended in sterile water and bound sequences were recovered with heat. Samples were then centrifuged and the supernatant was used in the next round as the enriched DNA pool. Counter selection was done by incubating an enriched pool with fibroblast cells, pelleting by centrifugation to partition bound sequences, and taking the supernatant (containing non-binding sequences) and directly incubating it with MA9Ras cells. Two counter selection rounds were done to eliminate off-target, non-specific binding following rounds 5 and 11. DNA pools collected after each round of selection were amplified and monitored by SYBR green qPCR. The cycle in which fluorescence can be detected is termed quantitation cycle (Cq for short) and is theresult generated by the thermocycler software following a qPCR experiment (representative traces are shown in Figure S2). A lower Cq value reflects higher initial copy numbers of the target, which is indicative of more binding sequences being recovered from the cell sample. The Cq values for a designated selection round are reported in Fig. 2. Increased library binding was indicated by an observed decrease in Cq number: the first round of selection yielded a Cq of 18.7 whereas in the final round of selection the Cq had decreased to 13.8. Other studies using qPCR have shown that a reduction in Cq values between selection rounds and an increase in the fluorescence signal is an indication of pool enrichment^61,62^. The consistency of the Cq value between thirteen and fourteen in the last three selections was encouraging evidencethat the pool had reached a maximum enrichment, and so the libraries were evaluated using high throughput sequencing following the 12th round.

### High throughput sequencing (HTS) and bioinformatics analysis

After twelve rounds of cell-SELEX, the resulting single strand DNA (ssDNA) library was sequenced using high throughput MiSeq Illumina sequencing. For detailed analyses see Figures S3–S5. The following selection rounds were sequenced: 0 (original random library), 6, 8, 11, 12, and 12 counter. The high throughput datasets were processed using AptaSUITE software^63^ which boasts several algorithms for aptamer sequencing analyses. The software generates several summary plots and values to gauge the success of the selection, including the total processed reads, the distribution of these reads between each pool analysed, the % base distribution of each pool analysed, and the unique fraction of each library. These data are summarized in Figure S3. Top sequence candidates were identified by sorting the sequencing data by highest count per million (Figure S4). Three sequences of interest were identified based on their high relative frequency compared to all other sequences, and their enrichment trends (Figure S4). Generally, in a sequencing analysis, if there is enough homogeneity of the enriched libraries clusters of aptamer families can be identified using the software. Though clustering failed for this selection due to the high fraction of the pool that was unique, a seven base consensus motif and its derivatives were identified in the top sequence candidates (Figure S5). Specifically, the count generated by AptaSUITE is the number of that sequence within a sequenced round normalized per million, whereas the enrichment is the ratio of the count from one sequenced round to the next. A high enrichment means that a given sequence appeared more often in the later round. Comparing enrichment of a sequence, and not just the copy number, from earlier to later selection rounds, allows for the choice of sequences with improved aptamer-target binding^64^. Full sequences of the three chosen aptamers can be found in Table 1 along with their measured apparent dissociation constant**.**

**Table 1:** Aptamer sequences of MLL-AF9 RAS target and their corresponding K_D_ values.

Aptamer	Aptamer sequence (5′–3′)	K_d_ (nM)
KGE01	TAGGGAAGAGAAGGACATATGATCACCACTCTTGAGTGGTGGATGAAGATGTATTGACTAGTACATGACCACTTGA	153.9 ± 98.2
KGE02	TAGGGAAGAGAAGGACATATGATCGCACACTATTAGAGTGTACGCATGATACATTGACTAGTACATGACCACTTGA	37.5 ± 2.5
KGE03	TAGGGAAGAGAAGGACATATGATGATGAGAGTACACATACTCTTGGATGACCATTGACTAGTACATGACCACTTGA	12.4 ± 2.2
Scrambled	ACAACGGTATGTTATGTATATCAATACTAACAGGTACAGTCGGATGATTCGAACATATCGGCGGTAGGAACACAGA	> 200

**Figure 3:**
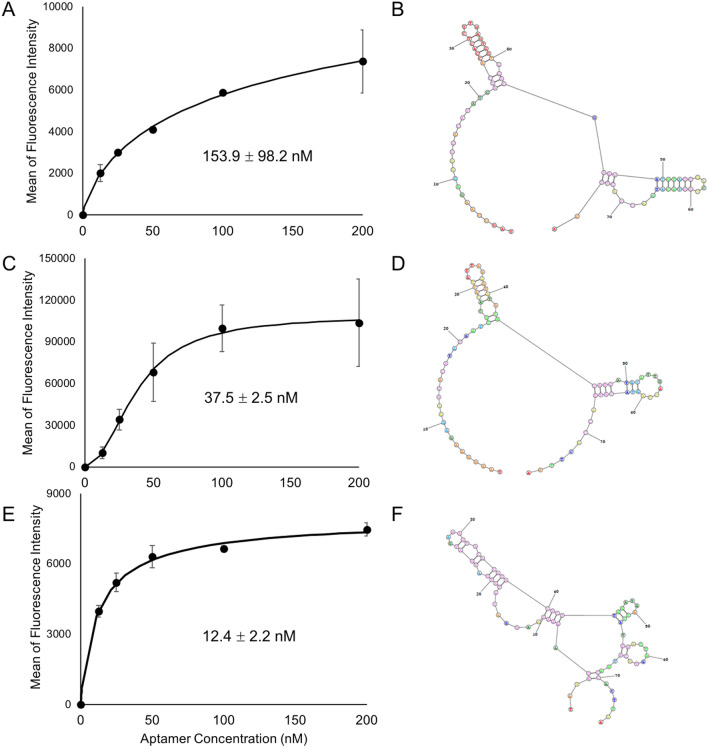
Binding curves of fluorescein labeled aptamers to MLL-AF9 RAS (MA9Ras) target cells. (**A**) KGE01 Kd curve: MA9Ras cells were incubated with increasing concentrations (nM) of KGE01 then were evaluated by flow cytometry. (**B**) The predicted secondary structure of KGE01 aptamer using RNA structure software. (**C**) KGE02 Kd curve: MA9Ras cells were incubated with increasing concentrations (nM) of KGE02 then were evaluated by flow cytometry. (**D**) The predicted secondary structure of KGE02 aptamer using RNAstructure software, a freely available web-based program that allows for the secondary structure prediction of single stranded DNA and RNA. (**E**) KGE03 Kd curve: MA9Ras cells were incubated with increasing concentrations (nM) of KGE03 aptamer using RNA structure software. To calculate the apparent Kd, the mean fluorescence intensity of the aptamer-cell dissociation vs concentration was fit to the equation Y = Bmax X/(Kd + X). The Kd graphs are the average of the three trials. Values are shown as means ± S.E.M.

The secondary structures and predicted DeltaG of all candidate aptamers were predicted using RNA structure software. All three candidates have relatively simple structures, with KGE01 and KGE02 having similar structures (Fig. 3B,D,F); however, the structure may be more complex when interacting with their target. DeltaG values for KGE01, KGE02, and KGE03 were − 12.8 kJ, − 6.8 kJ, and − 5.7 kJ, respectively. therefore, it is necessary to determine the binding constants to coincide with the predicted structures.

### Screening for the binding affinity of selected aptamers and K_D_ determination

To evaluate the binding affinity, the three selected aptamers were modified with a 5′ fluorescein tag and were subjected to binding studies via flow cytometry analysis against the target MA9Ras cell line to determine their apparent dissociation constant (K_D_) values. All K_D_ values obtained were in the nanomolar range, which is typical for aptamers developed against cancer cells using cell-SELEX^65^. Two of the selected aptamers, KGE03 and KGE02, showed K_D_ values in the low nanomolar range (12.4 + / − 2.2 nM and 37.5 + / − 2.5 nM, respectively) while the third, KGE01, showed a higher value (153.9 + / − 98.2 nM) (Fig. 3). KGE02 was chosen as the lead aptamer because of three qualities: the high enrichment between rounds, the nanomolar binding constant to the target, and the double hairpin secondary structure with the lower DeltaG, which allows for possible truncation. KGE02 showed the highest mean fluorescence intensity of the three aptamers and had a Hill Coefficient of 2, as compared to 1 for the other aptamers (fit equation data not shown). A Hill Coefficient greater than one indicates positive cooperativity in which the binding of one aptamer could facilitate the binding of subsequent aptamers. However, before further testing, it was necessary to first check the specificity of this particular sequence.

### Binding specificity of aptamers to cancer and normal cell cultures

**Figure 4:**
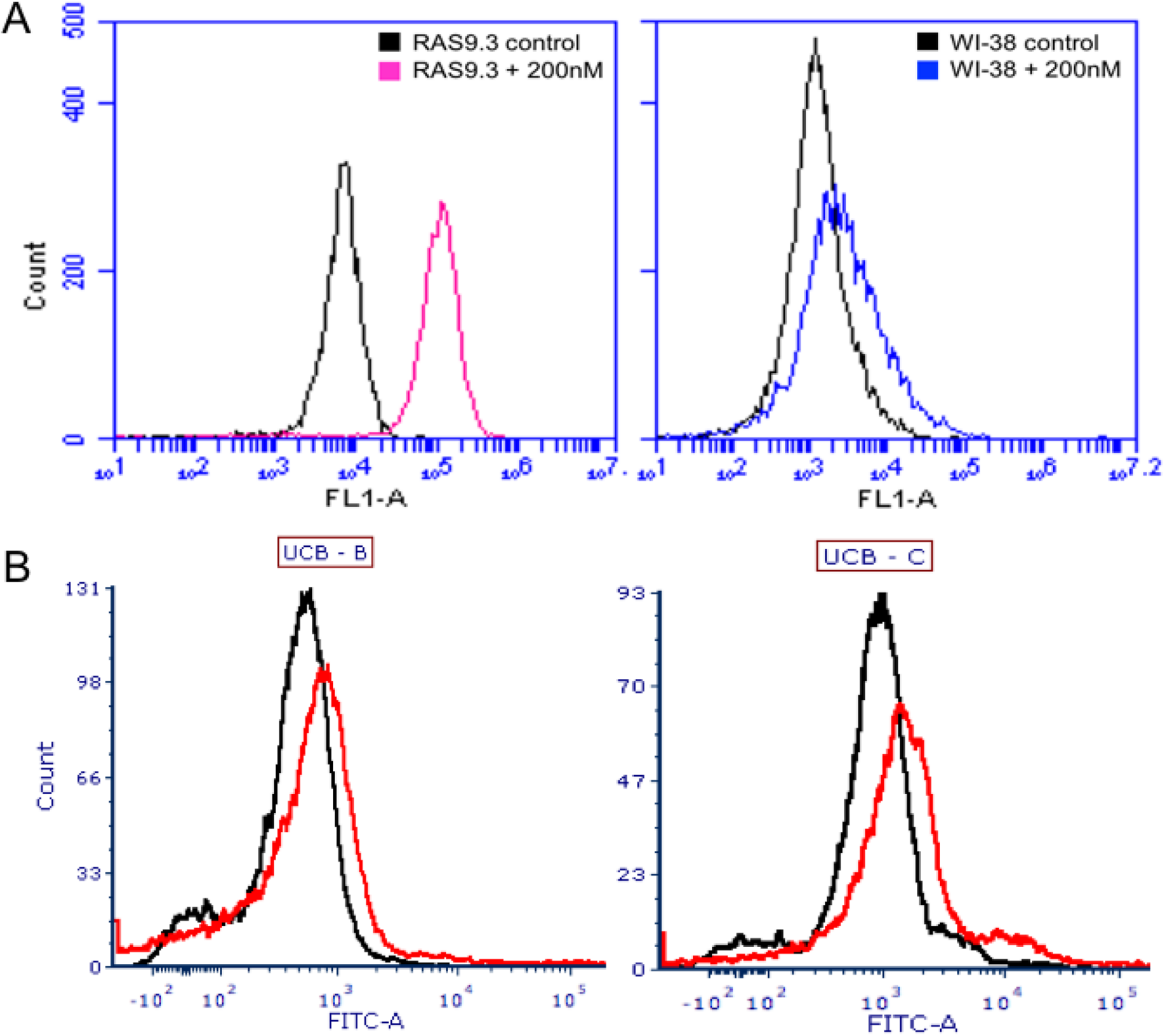
Binding of KGE02 aptamer to MLL-AF9 RAS (MA9Ras), WI-38, and UCB cells. (**A**) Mean fluorescence intensity of MA9Ras (left without (black) and with 200 nM KGE02 aptamer (pink); and mean of fluorescence intensity of WI-38 fibroblast (right) without (black) and with 200 nM KGE02 aptamer (blue). Cells were incubated and 10,000 events were counted by flow cytometry. Then data was analyzed using flow cytometry C6 sampler software. (**B**) Mean fluorescence intensity of two UCB cell lines without (black) and with 200 nM KGE02 aptamer (red). Cells were incubated and 10,000 events were counted by flow cytometry. Then data were analyzed using flow cytometry FCS Express software.

The binding specificity of KGE02 was further tested for normal and cancer cell lines via flow cytometry because of its predicted three-dimensional structure and low K_D_. The predicted structure had two central short hairpin loops flanked with 9 or more bases on either end, which would allow for potential truncation. As expected, KGE02 bound with a high affinity, shown by the shift in fluorescence, to the target MA9Ras cells (red line), but showed no affinity for WI-38, a normal human fetal lung fibroblast cells (blue line) (Fig. 4A). To further prove its specificity, KGE02 was incubated with two different lines of CD34 + human umbilical cord blood cell cultures. Results showed it had no affinity to either cell line (Fig. 4B). It is worth noting that the MA9Ras cell line was created by expressing the MLL/AF9 and NRAS(G12D) oncogenes in CD34 + UCB cells^58^.

**Figure 5:**
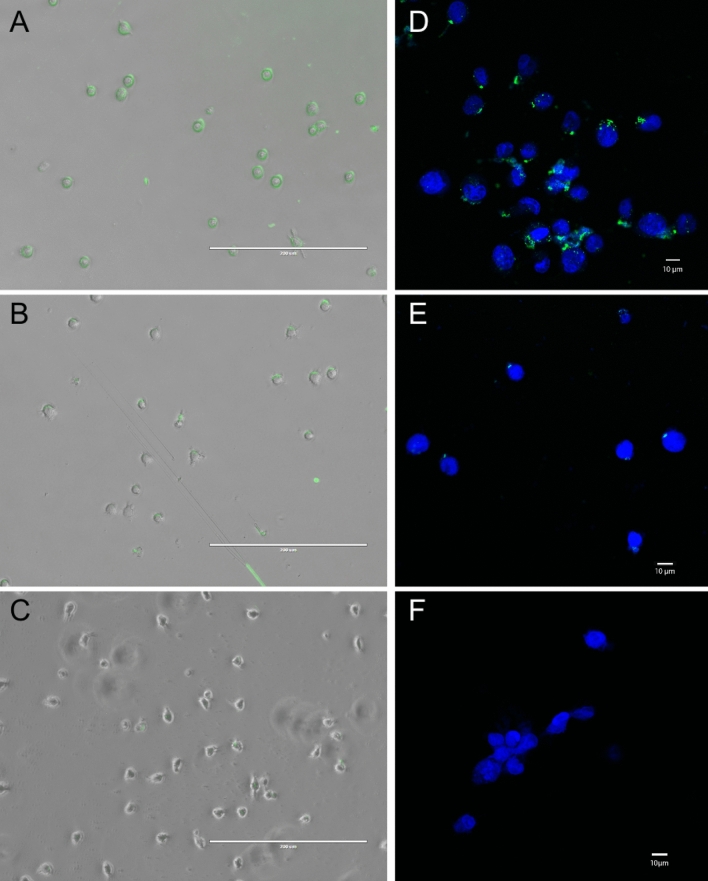
Localization of KGE02 to the cell membrane. (**A**) Overlay of brightfield and fluorescence microscopy images of 200 nM 6-FAM labelled KGE02 with MLL-AF9 cells. Clear localization of the fluorescence can be seen surrounding the cells. (**B**) Overlay of brightfield and fluorescence microscopy images of 200 nM 6-FAM labelled KGE02 with MLL-AF9 cells after DNA nuclease treatment. Most of the fluorescence signal has been lost after nuclease treatment, suggesting membrane localization of the aptamer’s target. (**C**) Overlay of brightfield and fluorescence microscopy images of 200 nM 6-FAM labelled scrambled sequence with MLL-AF9 cells. Minimal non-specific fluorescence can be detected. (**D**) Confocal microscopy image of 200 nM 6-FAM labelled KGE02 with MLL-AF9 cells stained with DAPI. Punctate localization of the fluorescence can be seen surrounding the cells. (**E**) Confocal microscopy images of 200 nM 6-FAM labelled KGE02 with AML-AF9 cells after DNA nuclease treatment. Most of the fluorescence signal has been lost after nuclease treatment, with minimal internalized fluorescence observed. (**F**) Confocal image of 200 nM 6-FAM labelled scrambled sequence with MLL-AF9 cells. No non-specific fluorescence can be detected.

We next used fluorescence and confocal microscopy to further confirm binding and gain some understanding of the localization of the cognate biomarker for KGE02. Fluorescein-tagged KGE02 was incubated with the cells and fluorescence images before and after a DNA nuclease treatment can be seen in Fig. 5. KGE02 shows distinct localization in the membrane of the MA9Ras cells (Fig. 5A). A scrambled version of the KGE02 sequence (Table 1) shows minimal binding at the same concentration (Fig. 5C), confirming that the interaction noted is more than non-specific oligonucleotide binding. After a DNA nuclease treatment of the KGE02-labelled cells, the majority of the fluorescence signal is lost (Fig. 5B), suggesting that the associated target for the aptamer is likely membrane-bound. We confirmed these observations by confocal microscopy. Once again, fluorescence from tagged-KGE-02 can be observed with a punctate localization surrounding the cells (Fig. 5D) which is mostly, but not completely, lost after DNA nuclease treatment (Fig. 5E). This fluorescence was notably absent when the cells are treated with tagged-scrambled sequence (Fig. 5F and Figure S6). Having determined its selectivity to the target and gathered some information about the potential location of the cognate target, the next step was to truncate to a shorter sequence to lower the cost of synthesis and to perhaps increase cell uptake in future applications.

### Truncation of KGE02 and assessment of their binding abilities

**Figure 6:**
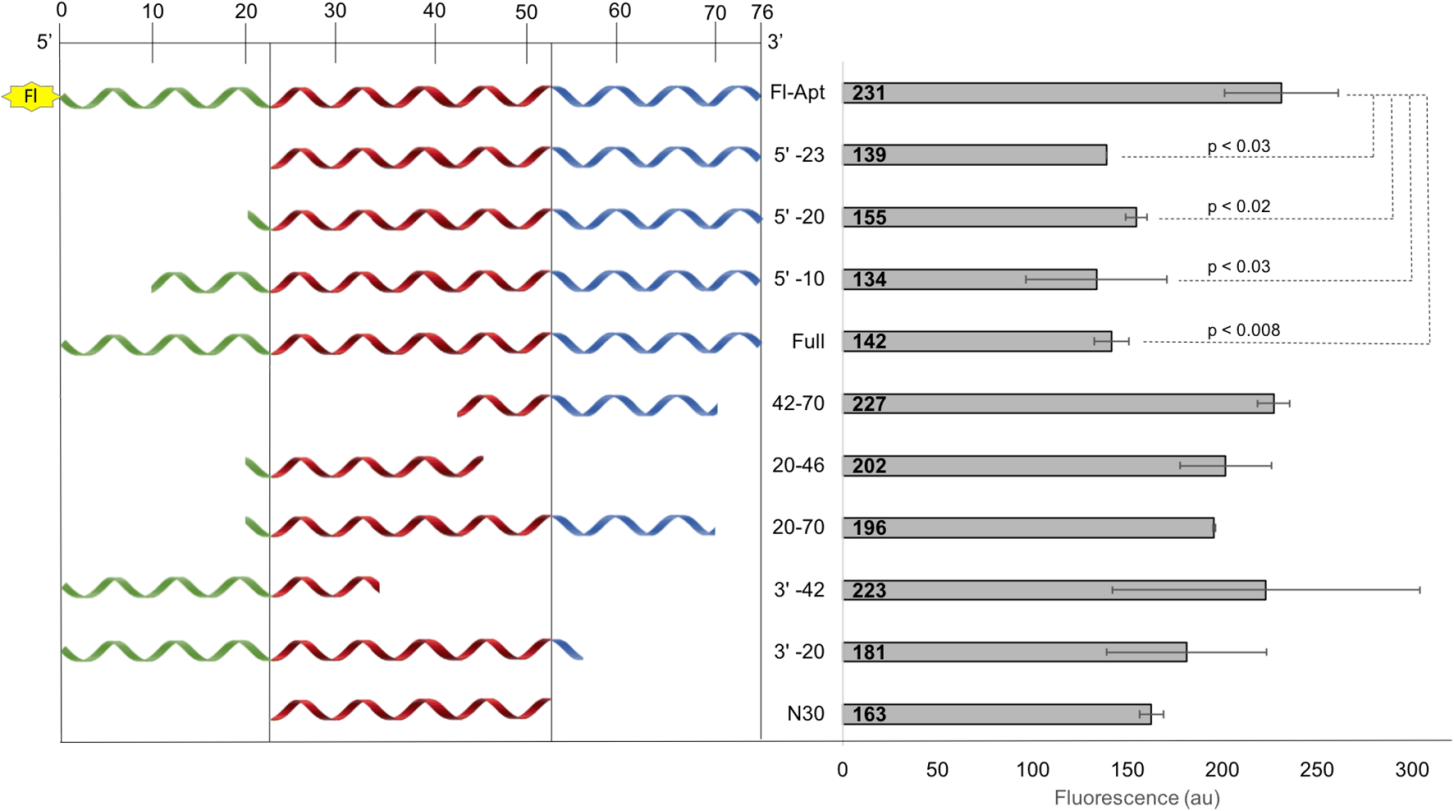
Truncation of KGE02 aptamer. Relative sequence length (left) and average fluorescence (right) of fluorescein-labeled full length KGE02, unlabeled full length KGE02, and nine unlabeled truncated sequences. Samples were incubated with truncated sequence (blocking sequence) for one hour then 200 nM fluorescein labeled KGE02 was added and samples were incubated for an additional hour. Reported p values are the truncated sequence fluorescence against the fluorescence of no truncated blocking sequence (Fl-Apt). Fluorescence was measured using a plate reader fluorimeter*.*

Once KGE02 was proven to be specific, the next step was to truncate the 76-base sequence to create a smaller binding sequence. Truncated sequences were created based on the predicted three-dimensional structure (Fig. 3B), which shows two hairpin loops formed between bases 20–70. Truncation from the 5′-end produced three sequences, in which either a portion (10 or 20 bases), or the entire 5′ primer domain (23 bases) was removed. Truncation from the 3′-end produced two sequences, in which either 20 bases (interrupting the second hairpin loop) or 42 bases (interrupting the first hairpin loop) were removed. Then, to determine if one or both hairpin loops were important to binding, three more sequences were produced. These sequences included both loops (20–70), just the first loop (20–46), and just the second loop (42–70). The final truncated sequence was the central region (30 bases) lacking both primers (24–54). Sequence lengths are listed in Fig. 6 (left side).

To determine binding ability, a blocking assay was performed. The premise of the assay was that if truncated sequences bound better than the 5′-fluorescein full length KGE02, the fluorescence signal would be decreased, based on competitive displacement. The larger the decrease in fluorescence, the greater the binding ability of the truncated sequence. MA9Ras cells were first incubated with 1 µM of the truncated sequence (non-fluorescent) for 60 min. Then, 200 nM of 5′-fluorescein tagged KGE02 (Fl-Apt) was directly added to the reaction and incubated for 60 min. Reactions were then pelleted, supernatant removed, resuspended in fresh buffer, and the total fluorescence of the solution was measured. As a maximum fluorescence control, MA9Ras cells were incubated with just 200 nM of 5′-fluorescein tagged KGE02; this gave the maximum fluorescence. As a minimum fluorescence control, MA9Ras cells were incubated first with 1 µM of full length non-fluorescent KGE02, followed by 200 nM of Fl-Apt; this gave the minimum fluorescence (maximum blocked). Results are quantified in Fig. 6.

Based on truncation results, it can be assumed that both hairpin loops are important in aptamer binding. Sequences that interrupted either loop (20–46, 42–70, 3′–20, and 3′–42) all showed little to no decrease in fluorescence. While the 30 base central domain lacking both primers showed a decrease in fluorescence, it was not statistically significant (*p* > 0.05) compared to the maximum fluorescence control from just Fl-Apt alone. Sequences that were truncated from the 5’ prime end showed the greatest blocking ability. Sequences 5′–10, 5′–20, and 5′–23 all showed significant decrease in fluorescence from just Fl-Apt alone and encroached toward the binding ability of the minimum fluorescence control. It can be concluded that both hairpin loops are necessary for aptamer binding and the 5′ primer is not important to the binding ability of the aptamer. To check that the removal of the 5′ primer did not change the predicted secondary structure, the structure of the shortened 53-base sequence was predicted using RNA structure software. The double hairpin structure in the full sequence remains uninterrupted in the shortened sequence, which can be seen in Figure S7.

### Binding of KGE02 to primary AML patient cells

**Figure 7:**
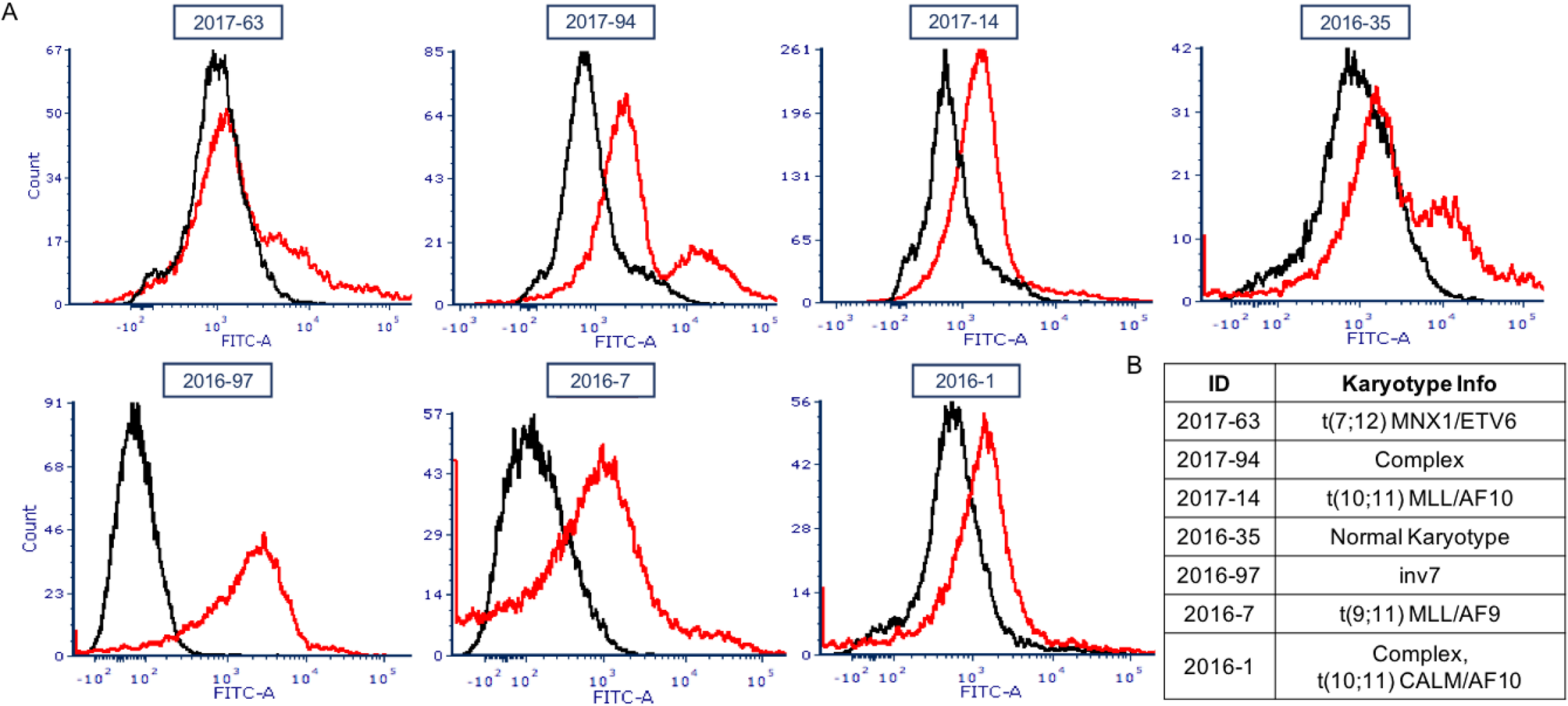
KGE02 binding to primary AML patient samples. (**A**) Mean of fluorescence intensity histograms of different AML primary patient samples (black) incubated with 200 nM KGE02 aptamer (red). Samples were incubated for an hour and 10,000 events were counted by flow cytometry. Data was analyzed using FCS Express software. (**B**) Table of the specific karyotype mutation of the primary patient samples.

5′-fluorescein labeled KGE02 aptamer was tested against seven primary AML patient cell lines for binding ability via flow cytometry analysis. The samples were from relapsed patients with a variety of different karyotypes. The binding screens were done at 200 nM, and the resulting histograms and karyotypes are shown in Fig. 7. Five of the seven samples showed binding (Samples 2017-94, 2017-14, 2016-97, 2016-7, and 2016-1), evidenced by a shift in fluorescence. The black line is the sample with no aptamer, and the red line is the sample incubated with aptamer. Samples 2017-63 showed no shift in fluorescence, and Sample 2016-35 showed very minimal binding, evidenced by the small shoulder peak.

There is a mix of karyotypes among the samples that show binding, and the two samples that did not show binding are not similar in karyotype. Samples 35 and 97 could be considered similar subtypes, called RAM immunophenotype, which was recently identified as a very aggressive type of AML^66,67^; the aptamer bound one RAM sample and not the other. This shows that there is no correlation to karyotype, or subtype of AML, and aptamer binding. However, it does show that the aptamer has been designed to bind a biomarker target on many AML subtypes, but the biomarker target does not appear on healthy umbilical cord blood cells that lack binding. Future work will involve efforts to characterize the molecular target of the aptamer^68,69^.

## Discussion

Using SELEX to produce aptamers that bind whole cells has been used in literature for a variety of targets. While there are some AML aptamers designed by whole-cell SELEX^70^, there is still a lack of published aptamers that bind multiple types of acute myeloid leukemia cells without using specific markers. Our technique and approach allowed for the development of a DNA library pool that binds MLL-AF9 RAS cells, proving that the whole-cell SELEX process using a cell line expressing a common oncogene was successful.

Characterization of cell-based aptamers relies strongly on flow cytometry, where one end of the aptamer is labeled with a fluorophore. High throughput sequencing of multiple selection rounds allows for the ability to compare enrichment and count between sequences. This allowed for the selection of three aptamers with different and unique sequences. The binding affinity of the three chosen aptamers was done via flow cytometry using a fluorescein tag, which is a common fluorophore and is commercially available. All three aptamers had binding constants in the nanomolar range (Table 1), so the secondary structures had to be considered. While KGE03 had the lowest binding constant, its secondary structure was complex, with multiple loops and junctures. KGE01 had a simpler structure but had the weakest binding. KGE02 had the desired low nanomolar binding constant 37.5 + / − 2.5 nM, and the secondary structure was not only simple (containing two hairpin loops that formed almost a key-like structure) but also had room on both the 5’-prime and 3’-prime ends for truncation (Fig. 3).

Prior to truncation, KGE02 was subjected to binding affinity studies. The counter selection in the SELEX process was fibroblast cells; therefore, it was necessary to test whether it showed any affinity. Fibroblast cells were used because of the desire to directly eliminate those sequences that disperse to organs or tissues not of interest in future clinical applications. While there was a slight shift in fluorescence, equating to some binding to fibroblast cells, the shift was not as evident as the one seen when the aptamer was incubated with the target MA9Ras cells. Even though the target cells were made by expressing leukemic oncogenes in a healthy CD34 + human umbilical blood cord cell cultures, KGE02 showed no apparent shift in binding, which means the biomarker responsible for binding is only expressed on AML cells (Figs. 4, 5).

Because the full-length aptamer was 76 bases long, a truncation study was done to try and make the sequence shorter, making it more cost effect and easier to manufacture. The secondary structure showed two hairpin loops, so truncation sequences consisted of sequences that cut the primers and the hairpin loops. Truncation of the 3’-prime end reduced its ability to bind, while loss of the 5’ primer still allowed the aptamer to bind (Fig. 6).

Because the goal is to be able to use the aptamer clinically, the affinity of KGE02 towards other subtypes of AML was tested. Five of the seven relapse or refractory AML samples showed some binding affinity, and there was no real correlation between binding and cell karyotype (Fig. 7). This indicates that KGE02 may effectively target a wide array of pediatric AML types and shows promise of future treatment with clinical drug conjugates.

## Materials and methods

### SELEX library and primers

The ssDNA library contained a 30-base randomized region flanked by 23 base PCR primer sequences (5′-TAGGGAAGAGAAGGACATATGAT-N30-TTGACTAGTACATGACCACTTGA-3′). Fluorescein labeled 5′ primer and poly-A 3′ primer were used during PCR. The ssDNA library was purchased from TriLink Biotechnologies (United States), and the primers were purchased from Eurofins Genomics (United States). The synthesized ssDNA was purified using 8% polyacrylamide gel electrophoresis.

### Cell culture

Umbilical cord blood (UCB) CD34 + cells were isolated with the EasySep CD34 + Selection Kit (StemCell Technologies). The MA9.3Ras cell line has been described previously^68^. Pre-existing and de-identified patient derived xenograft (PDX) cell lines were obtained from the Pediatric Avatar Program at Cincinnati Children's Hospital. MA9.3Ras AML cells were cultured in IMDM 20% bovine calf serum. Human umbilical cord blood (UCB) cultures and primary patient cell lines were cultured in IMDM 20% bovine calf serum with 1X Pen-Strep antibiotics and supplemented with 10 ng/mL of SCF, IL-3, IL-6, Flt-3L and TPO. Fibroblast cells were cultured in 5% Fetal Bovine Serum, 1X Antibiotic/Antimycotic Solution, 1 mM Sodium Pyruvate, 2 mM Glutamax, 10 ng/mL Epidermal Growth Factor, 5 µg/mL Insulin, 0.5 µg/mL Hydrocortisone in DMEM.

### Ethics

Human samples were acquired and used according to IRB protocols approved by the Cincinnati Children’s Hospital Institutional Review Board (Office for Research Compliance and Regulatory Affairs). De-identified UCB units were acquired by the Translational Trials Development Support Laboratory of Cincinnati Children’s Hospital after donating mothers provided informed consent (IRB #02-3-4x). De-identified PDX-derived leukemia cell lines were previously generated from diagnostic patient material originally obtained and utilized according to IRB protocols #2008-0021 and #2010-0658. Informed written consent of parents/guardians and assent of patient over 11 years old was obtained. All experiments were performed in accordance with relevant guidelines and regulations.

### Systematic evolution of ligands by exponential enrichment (SELEX) procedure (in vitro)

Positive selection: DNA pool (20–30 µL) was heated at 95 °C for 5 min. The pool was removed from the heat and Binding Buffer (10 mM Tris–HCl pH 7.5, 2 mM MgCl_2_, 140 mM NaCl) was added to 500 µL total volume, and then let to cool to room temperature. One million target cells were counted, pelleted, and washed twice with Binding Buffer. The cells were resuspended in the 500 µL solution containing DNA pool. Sample was incubated at 37 °C for 30 min, while shaking. The cells were pelleted and washed twice with Binding Buffer, then resuspended in 500 µL of sterile water. Cells were heated at 95 °C for 15 min to lyse. The supernatant was removed and used as next round DNA pool. For negative selection: DNA pool (20–30 µL) was heated at 95 °C for 5 min. The pool was removed from heat and Binding Buffer was added to 500 µL total volume, and then let to cool to room temperature. One million counter cells were counted, pelleted, and washed twice with Binding Buffer. The cells were resuspended in the 500 µL solution containing DNA pool, then incubated at 37 °C for 30 min, while shaking. The cells were pelleted, and the supernatant was removed. One million target cells were counted, pelleted, and washed twice with Binding Buffer. The supernatant was added to target cells and the positive selection protocol was finished.

### Quantitative polymerase chain reaction

Template Concentration Optimization: Each Round 1:10, 1:10, 1:1,000 dilutions were made with sterile water. Reactions (25 µL) were done with varying template volume (1–7 µL) of each dilution. Template concentration with the lowest measurable quantification cycle (Cq) was used in round amplification. Round Amplification: reactions were done on a 24-well PCR plate using a Thermo Scientific™ PikoReal™ Real-Time PCR System. Quantities listed are per reaction well: Maxima SYBR Green/ROX qPCR Master Mix 2X (Thermo Scientific) 25 µL and 2.5 µL of 10 µM (to a final concentration of 500 nM) primer. Template concentration and sterile water volume varied to total 50 µL per reaction well. 23 wells were complete reaction, 1 well was a no polymerase control. PCR of 40 cycles: denaturation of DNA at 95 °C for 30 s, annealing at 58 °C for 30 s, elongation at 72 °C for 45 s. Then a final elongation at 72 °C for 5 min. Quantification cycle was measured and obtained from the PikoReal™ software (Thermo Fisher, United States). Reactions were combined in an Eppendorf tube and dried with a speed vacuum overnight.

### Polyacrylamide Gel Electrophoresis (PAGE) Purification

Amplified SELEX rounds were purified via 8% PAGE. Gels were mixed as follows: 12 mL of 40% Acrylamide/Bisacrylamide, 6 mL 10X TBE, 42 mL deionized water, 500 µL 10% Ammonium Persulfate (APS), and 50 µL Tetramethylethylenediamine (TEMED). Gels were pre-run for 10 min at 12 W in 0.5× TBE. Sample was mixed 1:1 with denaturing loading dye (95% Formamide in 10× TBE with Bromophenyl Blue and heated at 95 °C for 10 min before loading. Gel was imaged, and fluorescent bands (5′-Fluorescein primer) were cut and suspended in water overnight, shaking. Gel was then filtered, and DNA was filtered through 3 k Molecular Weight Amicon Ultra-0.5 mL Centrifugal Filters. Add 100 µL of 10 mM Tris–HCl pH 7.5 to remaining DNA volume.

### Sequencing and structure prediction

High throughput sequencing (HTS) was performed using Illuminia sequencing. The enriched ssDNA pool from selection rounds 0, 6, 8, 11, 12+, and 12- SELEX were amplified via PCR using Illuminia special adapters TruSeq primers) to a minimum number of cycles (20 cycles). PCR products were purified using 8% PAGE and the DNA quantified using a Nanodrop spectrophotometer (Thermo Fisher, Canada). All amplified pools were combined to provide a total of 75 ng of DNA in each pool. Amplified pools were then sequenced at Carleton University using Illumina MiSeq sequencing platform. AptaSUITE^63^ (https://drivenbyentropy.github.io/) was used as the software to analyze the sequencing data and RNAstructure software (https://rna.urmc.rochester.edu/RNAstructureWeb/) was used to predict the secondary structure of the candidate sequences.

### Binding screen and K_D_ determination using flow cytometry

Binding tests were performed with 200 nM 5′-fluorescein tagged aptamer. Reactions contained 250,000 cells (MA9Ras or Fibroblast) in 250 µL of Binding Buffer (2 mM MgCl_2_-HBSS). Aptamer was heated at 95 °C in Binding Buffer for 5 min and cooled to room temperature before addition. Reactions were incubated at 37 °C for 30 min, shaking. Cells were pelleted and washed in 2 mM MgCl_2_-100 µg/mL BSA-HBSS. Samples were resuspended in 250 µL Binding Buffer and analyzed by flow cytometry. Flow studies were done on a BD FACSCanto at RFCC at Cincinnati Children’s Hospital. Samples were done in triplicate. K_D_ binding studies were done using 0, 12.5, 25, 50, 100 and 200 nM 5′-fluorescein tagged aptamer. Each reaction contained 250,000 cells (MA9Ras) in 250 µL of Binding Buffer (2 mM MgCl_2_-HBSS). Aptamer was heated at 95 °C in Binding Buffer for 5 min and cooled to room temperature before addition. Reactions were incubated at 37 °C for 30 min, shaking. Cells were pelleted and washed in 2 mM MgCl_2_-100 µg/mL BSA-HBSS. Samples were resuspended in 250 µL Binding Buffer and analyzed via flow cytometry. Flow studies were done on a BD FACSCanto at RFCC at Cincinnati Children’s Hospital. Samples were done in triplicate.

### Fluorescence and confocal microscopy

For preparing the slides for microscopy, coverslips were coated with poly-L-lysine (P4832) purchased from Sigma-Aldrich for 4 h at room temperature. Cells were grown on coverslips and treated with either 200 nM 6FAM-tagged KGE-02 or 6-FAM-tagged scrambled aptamer (5′-6-FAM-ACA ACG GTA TGT TAT GTA TAT CAA TAC TAA CAG GTA CAG TCG GAT GAT TCG AAC ATA TCG GCG GTA GGA ACA CAG A-3′).

After cell treatment with aptamer, cells were fixed with 4% paraformaldehyde for 10 min at room temperature, then washed with binding buffer. To remove the aptamer from the cell surface, the aptamer-coated cells were incubated in binding buffer containing 2.5Unit/μL DNase I, amplification grade (AMPD1), (Sigma-Aldrich) at 37 °C for 10 min. In all treatments, the cells were washed with the binding buffer and were imaged by fluorescence microscopy using an EVOS FL fluorescence microscope with a 40 × objective. Cells were imaged on both brightfield and GFP filters, and the images merged.

For the confocal experiments cells were also stained for 15 min at room temperature with PureBlu DAPI Nuclear Staining Dye (Bio-Rad, Hercules, California) followed with washing the coverslips with 1 × PBS. Then we applied the appropriate volume of mounting medium and sealed the coverslip with nail polish and observed them under the confocal microscope. Samples were run in triplicate. High magnification confocal photomicrographs were acquired using a Nikon C2 confocal system (Nikon Instruments Inc., Mississauga, Canada) on an Eclipse Ti2 inverted microscope (Nikon) with a Plan Apochromat 40 × objective lens (0.95 numerical aperture). Cells were excited using lasers with excitation lights of 405-nm and 510-nm wavelength to visualize 4′,6-diamidino-2-phenylindole (DAPI) and fluorescein-labeled aptamers, respectively. All images were acquired with identical laser power settings. Images were processed with NIS-Elements software (Nikon Corporation, Konan, Japan) so that DAPI and fluorescein-labeling were pseudocolored blue and green, respectively. Image brightness and contrast were adjusted for each channel via the range of “lookup table” (LUT) values. The LUT range established for DNase-treated cells were applied to images from all treatment conditions to enable an accurate comparison of localization and fluorescence intensity between cell treatment conditions.

### Truncation study

Truncation studies were done as a fluorescence blocking assay. Reactions contained 500,000 cells (MA9Ras) in 250 µL of Binding Buffer (2 mM MgCl_2_-HBSS). All aptamer sequences were heated at 95 °C in Binding Buffer for 5 min and cooled to room temperature before addition. The first incubation was done with 1 µM of non-fluorescent blocking sequence or no aptamer at room temperature for 60 min, shaking. After 60 min, 200 nM 5′-fluorescein tagged aptamer was added directly to all samples for the second incubation at room temperature for 60 min, shaking. Cells were pelleted and washed in 2 mM MgCl_2_-100 µg/mL BSA-HBSS. Samples were resuspended in 250 µL Binding Buffer and analyzed by a fluorimeter with a plate reader. Samples were done in triplicate. P values were calculated using Kaleidagraph*.*

### Primary patient binding studies using flow cytometry

Binding tests were done with 200 nM 5′-fluorescein tagged aptamer. Reactions contained 250,000 cells (cultures derived from 7 AML patient samples at CCHMC) in 250 µL of Binding Buffer (2 mM MgCl_2_-HBSS). Aptamer was heated at 95 °C in Binding Buffer for 5 min and cooled to room temperature before addition. Reactions were incubated at 37 °C for 30 min, shaking. Cells were pelleted and washed in 2 mM MgCl_2_–100 µg/mL BSA-HBSS. Samples were resuspended in 250 µL Binding Buffer and analyzed by flow cytometry. Flow studies were done on a BD FACSCanto at RFCC at Cincinnati Children’s Hospital Medical Center.

## Supplementary Information

Supplementary Information.

## Abbreviations

AML: Acute myeloid leukemia
MLL-AF9: Mixed lineage leukemia AF9 fusion protein
SELEX: Systematic evolution of ligands by exponential enrichment
DDS: Drug delivery system
PTK7: Protein tyrosine kinase 7
CD117: (KIT) Type III receptor tyrosine kinase
MA9Ras: MLL-AF9 NRas cell line
qPCR: Quantitative polymerase chain reaction
Cq: Quantification cycle
HTS: High throughput screening
ssDNA: Single strand DNA
KGE##: Sequenced aptamers
K_D_: Dissociation constant
WI-38: Normal human fetal lung fibroblast cells
CD34+: Hematopoietic stem cell expressing glycoprotein CD34
UCB: Umbilical cord blood cells
Fl-Apt: 5′-Fluorescein tagged 76-base KGE02 aptamer
RAM: Immunophenotypic subtype of AML
