# Supplementary material for: Development and characterization of a DNA aptamer for MLL-AF9 expressing acute myeloid leukemia cells using whole cell-SELEX
Source: Sci Rep. 2021 Sep 27;11:19174. doi: 10.1038/s41598-021-98676-4 (PMC8476576; doi:10.1038/s41598-021-98676-4)
Supplement: Supplementary file 1 — Supplementary Information. [file 41598_2021_98676_MOESM1_ESM.docx]

**Supplementary Information**

**
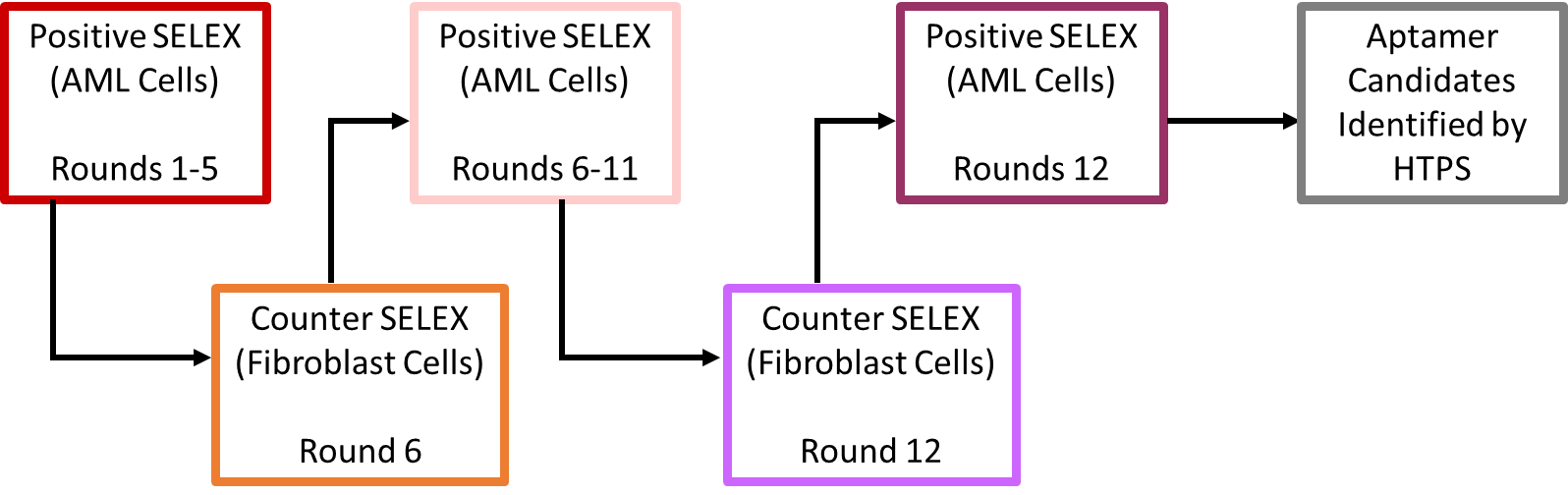
**

Figure S1: Workflow schematic of positive and counter selection rounds performed during the selection of aptamers for AML cells. HTPS: high throughput sequencing.

**
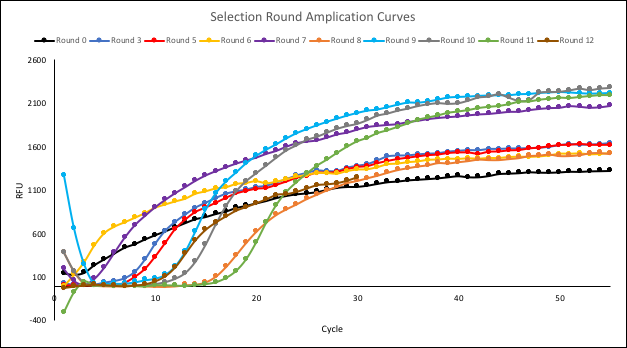
**

Figure S2: Quantitative PCR amplification plots for SELEX Rounds used to determine enrichment by round. Cq values were calculated by the thermocycler software based on the cycle where the detected fluorescence was measured above the set detection threshold.


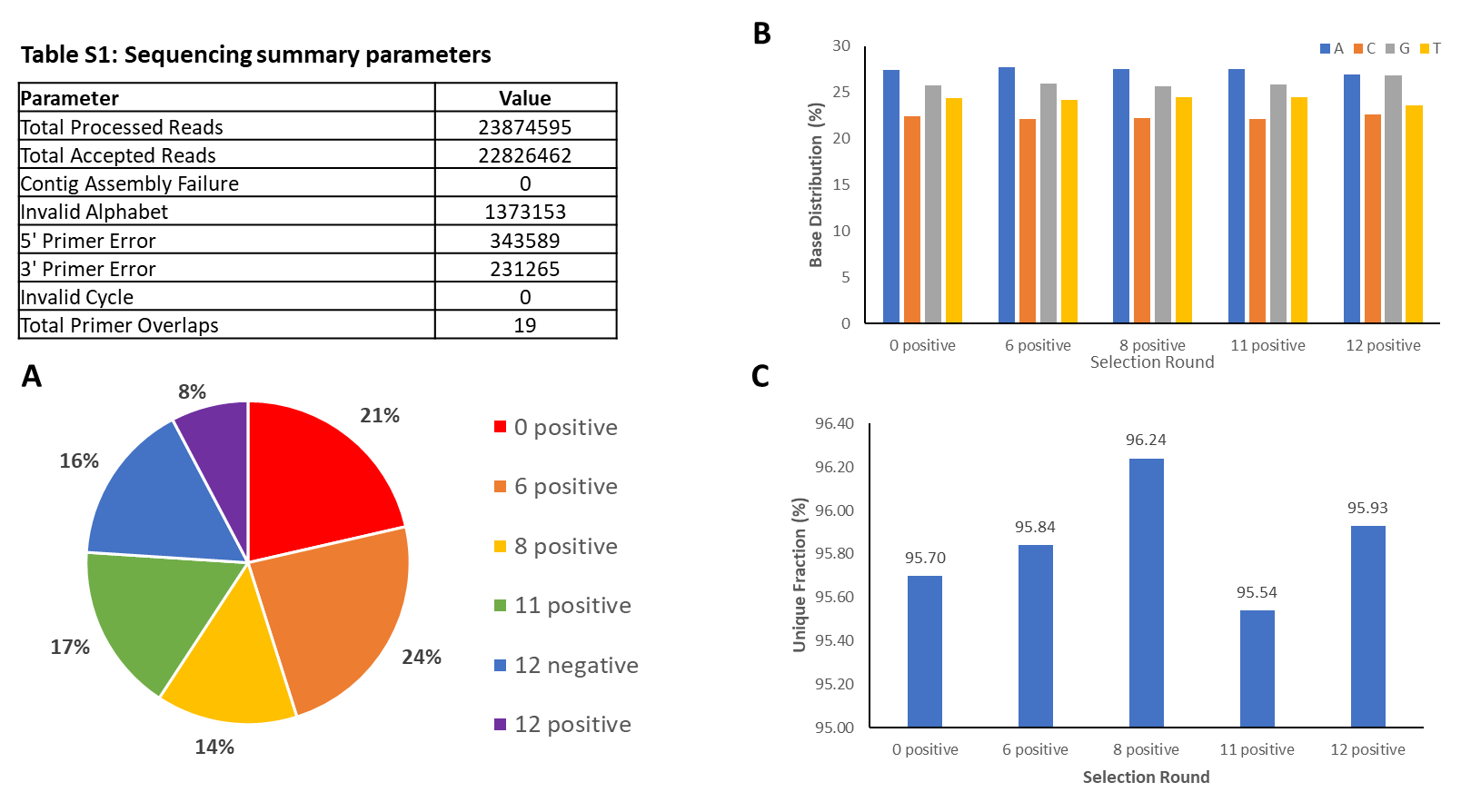

Figure S3: Selection pool sequencing was accomplished using AptaSUITE, which is freely available at <https://drivenbyentropy.github.io/>. Table S1 summarizes the sequencing parameters, demonstrating that the number of sequences analyzed for the total experiment was in the millions. The distribution of the reads per round is shown in panel A. The library started out, and maintained a relatively equal base distribution (panel B) throughout the selection, indicating that the library was not skewed towards a particular base, as one might see if the most competitive aptamers were G-quadruplexes. Finally, panel C shows the fraction of the library that had unique sequences per selection round. Typically the unique fraction of the selection library would decrease with increasing selection rounds, however a distinct advantage of high throughput sequencing is that high affinity aptamers can be identified earlier in selections than when enrichment indicators necessarily hit a plateau. More information about sequencing analysis can be found by reading: AptaSUITE: A Full-Featured Bioinformatics Framework for the Comprehensive Analysis of Aptamers from HT-SELEX Experiments. Hoinka, J., Backofen, R. and Przytycka, T. M. (2018). Molecular Therapy - Nucleic Acids, 11, 515–517. <https://doi.org/10.1016/j.omtn.2018.04.006>


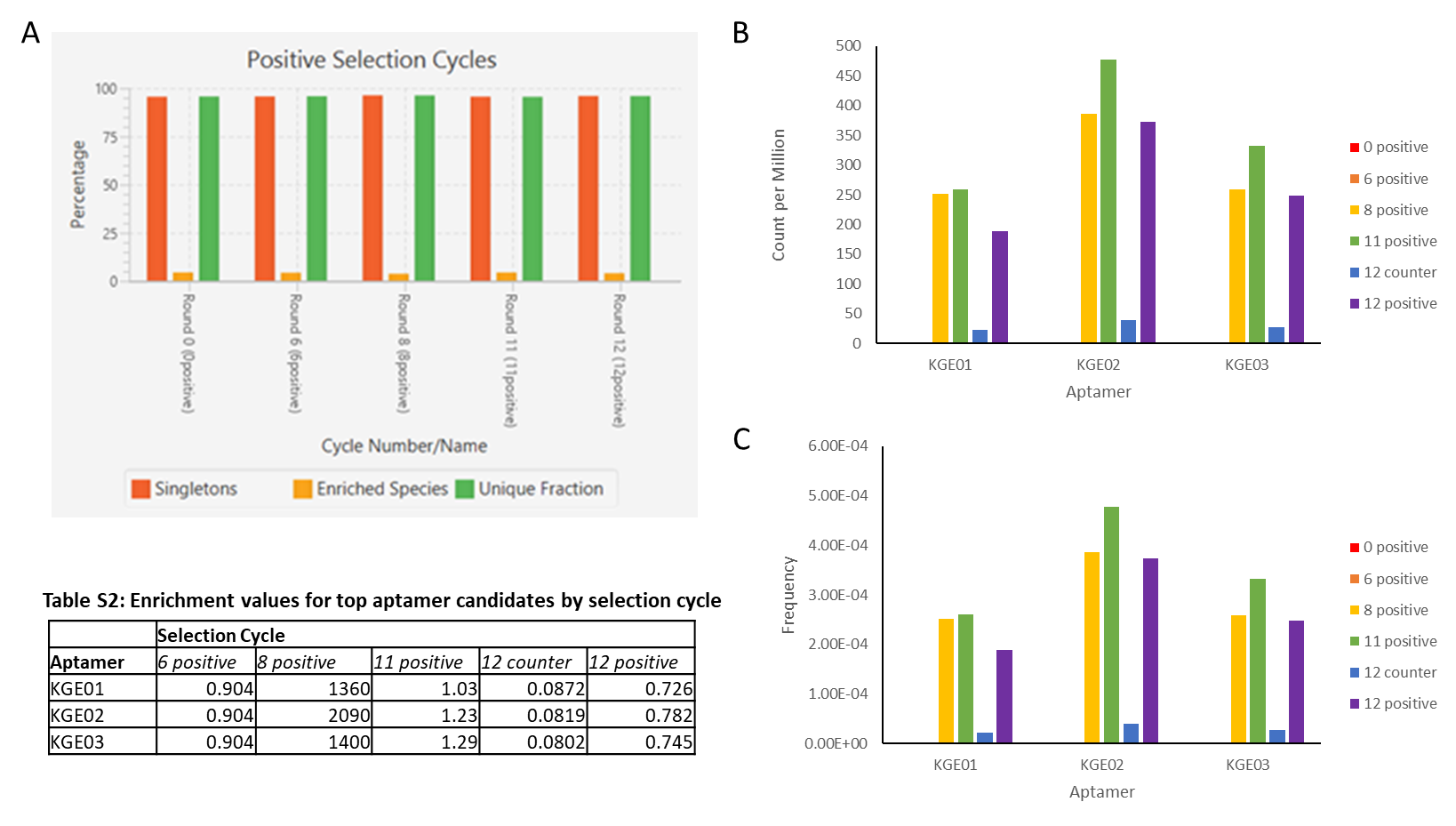

Figure S4: Summary of enrichment, count, and frequency data from the high throughput sequencing. A) screen capture image of the summary chart produced by AptaSUITE (for more details on AptaSUITE, see the caption of Figure S3). The sequencing pools consisted of mostly unique sequences. The enrichment data is summarized in table S2. Considerable enrichment of the top three sequences was observed in the 8^th^ positive selection cycle. Likewise, the count (panel B) and frequency (panel C) of the aptamer candidates also increased largely in the 8^th^ positive selection cycle.


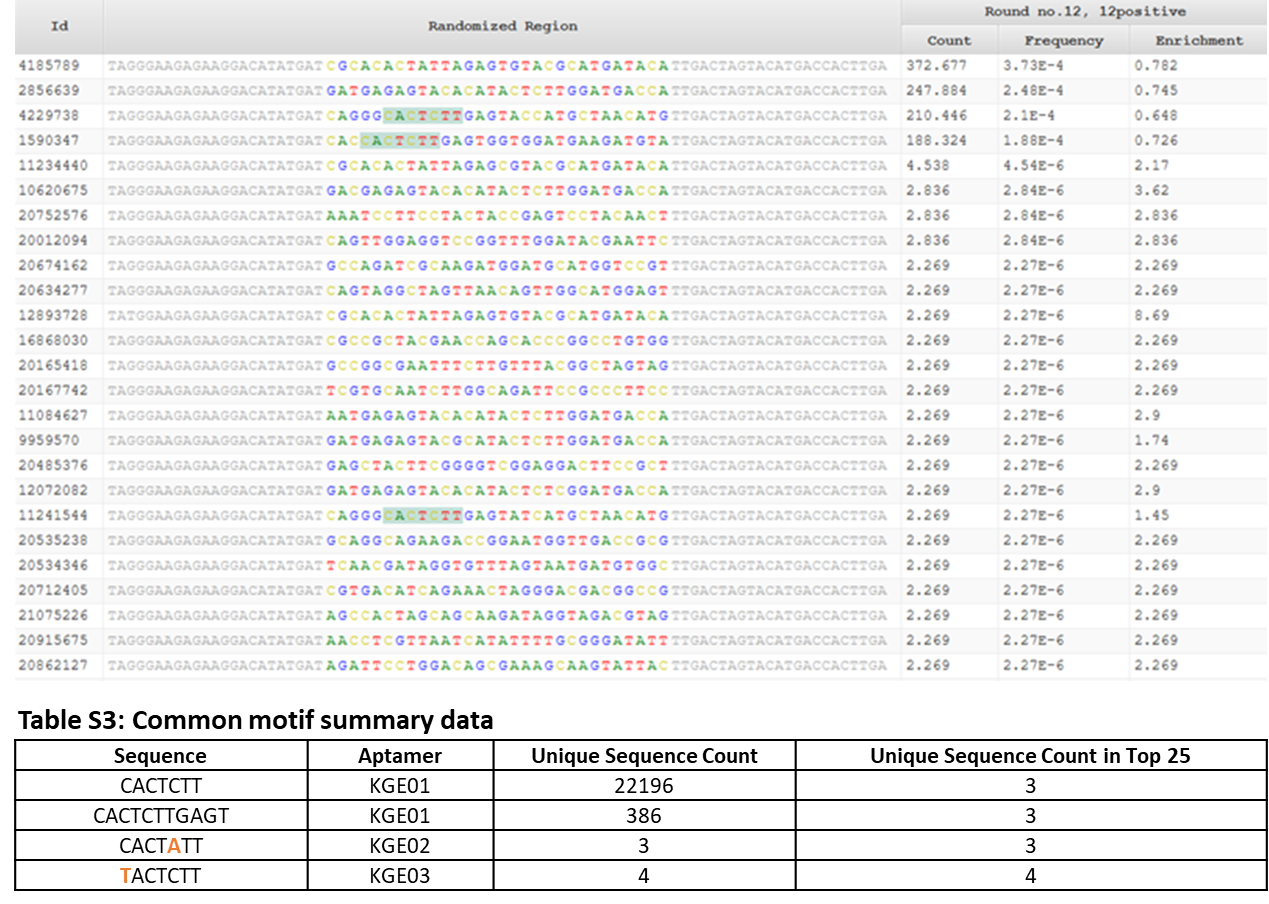


Figure S5: Representative sequence information (top) and common motif summary data (Table S3) for the CACTCTT motif and its variants identified in the top sequence candidates. The top image is a screen capture of the top 25 sequences and the presence of the most common motif identified: CACTCTT generated by AptaSUITE (see details in Figure S3 caption). The CACTCTTGAGT sequence was extended compared to the dominant motif and existed in KGE01. The other two motifs contained single point mutations (colored orange) of C🡪A (KGE02) and C🡪T (KGE03) compared to the dominant motif. Interestingly, the motifs were nearly equally represented in the top 25 sequences (sorted by count in descending order) despite the abundance of the CACTCTT motif in the selection pool.


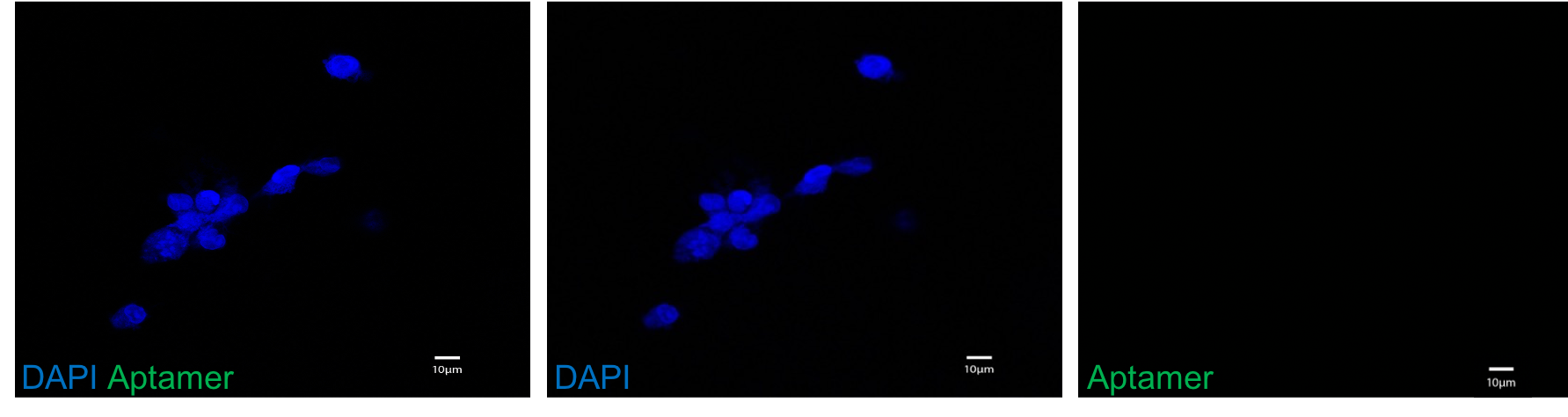


Figure S6: Individual Confocal Microscopy Images of Scrambled Sequence with MLL-AF9 cells. A) Scrambled (200 nM) and DAPI (50 μl) without nuclease treatment, OVERLAY. B) Scrambled (200 nM) and DAPI (50 μl ) without nuclease treatment, DAPI channel. C) Scrambled (200 nM) and DAPI (50 μl ) without nuclease treatment, fluorescent channel.


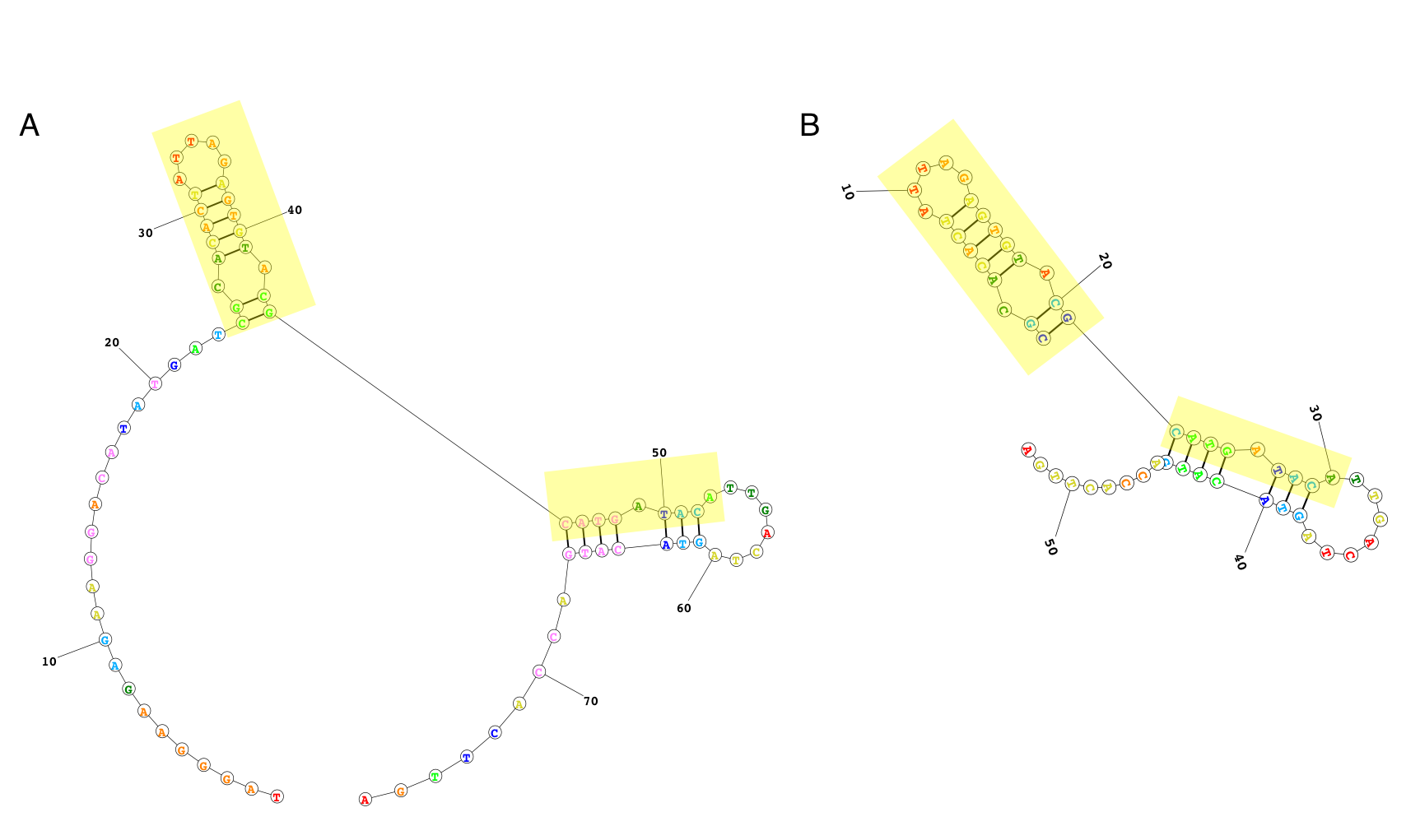


Figure S7. Comparison of secondary structures of KGE02 with full length or truncated sequence. (A) The predicted secondary structure of the full length KGE02 aptamer using RNAstructure software. (B) The predicted secondary structure of the KGE02 aptamer lacking the 23-base 5’primer using RNAstructure software. The bases highlighted in yellow are the 30 sequenced bases from the N30 region*.*
